# Errors in the Calculation of 27Al Nuclear Magnetic Resonance Chemical Shifts

**DOI:** 10.3390/ijms131115420

**Published:** 2012-11-21

**Authors:** Xianlong Wang, Chengfei Wang, Hui Zhao

**Affiliations:** 1 Center of Bioinformatics, University of Electronic Science and Technology of China, No. 4, 2nd Section, Jianshe Road, Chengdu 610054, China; E-Mails: chengfeiwang.aka.chance@gmail.com (C.W.); kathy517413@sohu.com (H.Z.); 2 Department of Chemistry, Bryn Mawr College, 101 North Merion Avenue, Bryn Mawr, PA 19010, USA

**Keywords:** aluminum-27 NMR, aluminum(III) complexes, computational chemistry, density functional theory

## Abstract

Computational chemistry is an important tool for signal assignment of ^27^Al nuclear magnetic resonance spectra in order to elucidate the species of aluminum(III) in aqueous solutions. The accuracy of the popular theoretical models for computing the ^27^Al chemical shifts was evaluated by comparing the calculated and experimental chemical shifts in more than one hundred aluminum(III) complexes. In order to differentiate the error due to the chemical shielding tensor calculation from that due to the inadequacy of the molecular geometry prediction, single-crystal X-ray diffraction determined structures were used to build the isolated molecule models for calculating the chemical shifts. The results were compared with those obtained using the calculated geometries at the B3LYP/6-31G(d) level. The isotropic chemical shielding constants computed at different levels have strong linear correlations even though the absolute values differ in tens of ppm. The root-mean-square difference between the experimental chemical shifts and the calculated values is approximately 5 ppm for the calculations based on the X-ray structures, but more than 10 ppm for the calculations based on the computed geometries. The result indicates that the popular theoretical models are adequate in calculating the chemical shifts while an accurate molecular geometry is more critical.

## 1. Introduction

Studies with aluminum(III) (Al(III)) complexes are important in bioinorganic chemistry, geochemistry, environmental science, and material science due to the toxic effects of many aluminum compounds and their industrial usage as catalysts and coagulation agents. Because various kinds of species of Al(III) might differ significantly in their toxic effects, speciation of aqueous Al(III) complexes is of great value to study their biological and environmental roles [1,2]. Nuclear magnetic resonance spectroscopy (NMR) has been routinely used in studying the coordination chemistry and speciation of Al(III) in aqueous solutions due to its noninvasive character [3].

^1^H, ^13^C, ^17^O and ^27^Al are commonly used nuclei for observing NMR spectra of Al(III) complexes. Among them, ^27^Al possesses advantages over the others, including 100% abundance, high signal sensitivity, a wide range of chemical shifts, and sensitivity to coordination environment. For example, the ^27^Al chemical shift (δ_Al_) of the tetrahedral [Al(OH)_4_]^−^ species is 80 ppm relative to the octahedral [Al(OH_2_)_6_]^3+^ species. Therefore, solution-state ^27^Al NMR spectroscopy has been a very powerful tool to characterize the structures of Al(III) complexes, to monitor the hydrolysis of Al(III), to identify Al(III) species in biological and environmental samples, and to quantify the concentrations of Al(III) species [3]. It is particularly useful for the characterization of those species which are difficult to isolate and/or to crystallize or inert for other spectroscopic methods.

^27^Al nucleus has a spin of 5/2 and with a moderately large electric quadrupole moment. Both the chemical shift and line width of ^27^Al signals are sensitive to aluminum coordination geometry. For a highly asymmetric coordination geometry, the resonance peaks of ^27^Al may be too broad to observe. In aqueous solutions, the coordination number of Al^3+^ monomers fluctuates between 4 and 6. Many aqueous Al(III) complexes are octahedral-coordinated and some may be distorted. As a result, δ_Al_ of these complexes are not far away from 0 ppm relative to the aqueous [Al(OH_2_)_6_]^3+^ species and the chemical shift spectra peaks are not sharp. Furthermore, due to the tendency of Al(III) to hydroxylize, to oligomerize and to form mixed complexes, the solutions are often a complicated system with many coexisting species. Often, many ^27^Al chemical shift signals are crowded together in the vicinity of 0 ppm. Although tentative structural information can be made by comparing the spectroscopic features of the unknown species with model complexes, it is usually difficult to make unique assignments. Therefore, computational modeling, along other experimental methods, is often used to complement ^27^Al NMR experiments.

With the rapid development of computational chemistry, its application has become almost ubiquitous in all branches of chemistry. In the study of Al(III) complexes, computational methods have been used to predict NMR spectra. For example, Tossell [4] calculated the chemical shieldings of ^27^Al, along with other nuclei, by applying the gauge-including atomic orbital (GIAO) method [5,6] with the Hartree-Fock (HF) theory and to elucidate the species formed during the hydrolysis and the oligomerization process of Al(III) in aqueous solution. Amini *et al.*[7,8] applied the HF theory and density functional theory (DFT) to calculate the solid-state ^27^Al NMR spectra of aluminum acetylacetonate and other similar complexes for the clarification of the crystal structure of the compounds. Mirzaei *et al.*[9,10] used DFT calculations to calculate ^27^Al and ^31^P chemical shieldings in the investigation of aluminum phosphide and nitride nanotubes. The gauge including projected augmented wave (GIPAW) method [11] developed by Mauri and Pickard in 2001 [12] which enabled the calculation of all-electron NMR parameters in solids was also used to predict the solid-state ^27^Al NMR spectra [13,14].

Although the calculations of chemical shieldings have played an important role in the determination of structures, there have been a limited number of systematic studies concerned with the accuracy of computational models that predict the chemical shifts of ^27^Al. Mulder *et al.*[15] reviewed several theoretical methods for the calculation of NMR chemical shifts and they evaluated the applications of these methods in protein structure determination. Jensen [16] evaluated the basis set convergence problem in calculating NMR shielding constants calculated by two DFT methods and proposed to use pcS-*n* basis sets for calculating shielding constants. In 1999, Kubicki *et al.*[17] performed a thorough computational study on Al(III) hydrolysis products and Al(III)-carboxylate complexes to determine how accurately the ^27^Al chemical shifts can be calculated using *ab initio* methods. The study was based on the self-consistent isodensity polarized continuum model (SCIPCM) of the aqueous species using HF and second-order Møller-Plesset perturbation (MP2) theories. By comparing the calculated chemical shifts with the experimental values, they suggested that the ^27^Al NMR spectra should be reinterpreted in some previous publications. Ten years later, Bi *et al.*[18] assessed the accuracy of ^27^Al chemical shift calculations that used 29 DFT functionals, HF and MP2 models. They concluded that of all the models, the HF and MP2 models gave the most accurate results. Their conclusion was made on the basis of the calculations of only a few Al(III) hydrolysis products. It remains unanswered if the conclusion holds for more general Al(III) complexes.

Previous studies found that the error involved in the *ab initio* calculation of ^27^Al chemical shifts is typically approximately 10 ppm and for some DFT models it could be as large as 30 ppm [18]. Errors of such magnitude pose serious limitations for the application in assigning experimental spectra, since the ^27^Al chemical shifts of many octahedral-coordinated complexes are typically distributed in the small range from −10 to 30 ppm. To distinguish between the small differences found in similar complexation species, a computational method must be able to reproduce the chemical shifts with an error within a few ppm and at least be able to put the resonance peaks in the correct order.

There are two major parts to the errors in calculated ^27^Al chemical shifts: the error in the prediction of the geometry and the error in the chemical shielding determination. Previous studies presenting the calculations of ^27^Al chemical shifts did not differentiate between these two kinds of errors. There have been many publications concerning the accuracy of *ab initio* models including those that use DFT in predicting complex geometries [19]. The purpose of this work is to evaluate the performance of several commonly used theoretical models, including HF, MP2 and DFT, in predicting the chemical shifts of Al(III) complexes. Our approach is to use the X-ray crystallographic structures to calculate the chemical shifts and then to compare them with the experimentally measured values. It has been well established that X-ray diffraction structures represent well the molecular geometries in solutions except for the rotation-flexible functional groups.

## 2. Results and Discussion

More than 200 single-crystal X-ray diffraction determined geometries [20–145] of 114 Al(III) complex species were obtained from the Cambridge Crystal Database. (There is more than one crystal structure available for some complexes.) In most species, Al(III) is found in an octahedral-coordination environment binding with six ligands or functional groups. Some Al(III) are found in tetrahedral coordination environments and a limited number of species are in penta-coordination environments. Usually Al(III) binds with ligands via O atoms. Al–N ranks second in the binding modes. A few species of Al(III) coordinated with fluorides and phosphates were also examined. This covers most coordination forms commonly encountered in the study of solution aluminum chemistry. Other coordination forms, such as planar tri-coordination and complexation with other ligands, e.g., H and Cl, were not studied in this work.

(1) $$\sigma_{\text{A}1}\;(\text{GIAO}-\text{B}3\text{LYP}/6-31\text{G}(\text{d}))=114.1+0.85\;\sigma_{\text{A}1}\;(\text{GIAO}-\text{B}3\text{LYP}/6-311+\text{G}(\text{d},\text{p})),R^{2}=0.95$$

(2) $$\sigma_{\text{A}1}\;(\text{GIAO}-\text{HF}/6-311+\text{G}(\text{d},\text{p}))=59.4+0.96\;\sigma_{\text{A}1}\;(\text{GIAO}-\text{B}3\text{LYP}/6-311+\text{G}(\text{d},\text{p})),R^{2}=0.98$$

### 2.1. Chemical Shielding Constants

The chemical shielding tensors of each structure were calculated with the GIAO formalism [5,6] at the following four popular model levels, B3LYP/6-31G(d), HF/6-31G(d), B3LYP/6-311+G(d,p) and HF/6-311+G(d,p). In addition, MP2/6-311+G(d,p) was also applied to species of [Al(OH_2_)_6_]^3+^, [AlF_6_]^3−^ and [AlF_5_(OH_2_)]^2−^.

**Figure 1:**
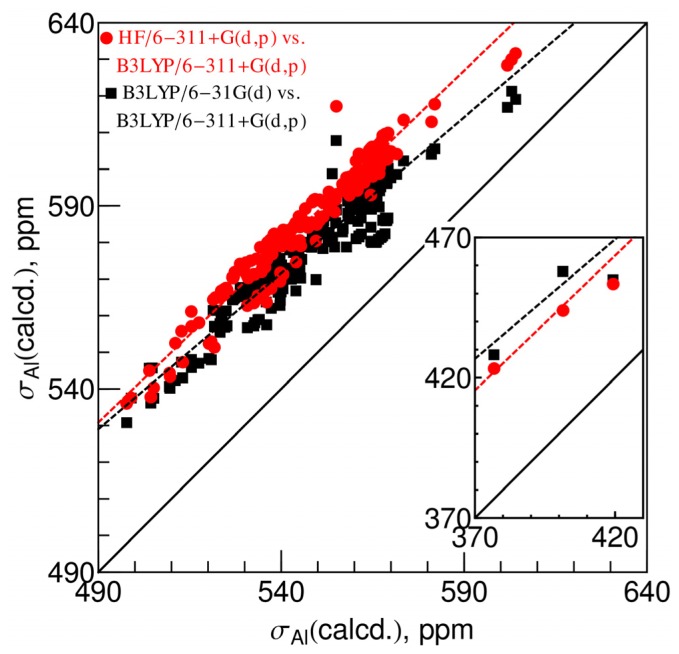
Correlation between the calculated isotropic ^27^Al chemical shielding constants using the X-ray crystallographic geometries at the B3LYP/6-31G(d) (black squares) and the HF/6-311+G(d,p) (red circles) levels *versus* those obtained at the B3LYP/6-311+G(d,p) level. The black dashed line and the red dashed line are the best-fit linear regression of the two data sets, respectively, and the best-fit equations are given in Equations 1 and 2. The solid black line is the ideal diagonal line (*y* = *x*).

Figure 1 shows how the calculated isotropic shielding constants at different levels are correlated. The *x*-axis represents the values calculated at the B3LYP/6-311+G(d,p) level. On the *y*-axis direction, the shielding constants obtained at the B3LYP/6-31G(d), and HF/6-311+G(d,p) levels are represented as black squares and red circles, respectively. There is a strong linear correlation in a wide range from 350 to 600 ppm for both data sets, though the chemical shieldings calculated at different levels of theory could differ as large as 60 ppm. In general, the hybrid B3LYP functional gives consistently smaller shielding constants than the HF theory and so does the larger basis set (6-311+G(d,p)) than the smaller (6-31G(d)) basis set.

Comparing the two differently sized basis sets, 6-31G(d) *versus* 6-311+G(d,p), both using the B3LYP model, the coefficient of determination (*R*^2^) of the linear regression is 0.95. The best-fit straight line is Equation 1 (black dashed line in Figure 1). The majority of the absolute fitting residuals (74%) are less than 5 ppm. There is one outlier point with a fitting residual of 24 ppm, while all the rest are less than 13 ppm. The outlier point is an Al-phosphate species. The exact reason for the outlier is unclear.

The shielding constants obtained at the HF/6-311+G(d,p) level are slightly higher than those at the B3LYP/6-31G(d) level. They also have strong linear correlations with the values obtained at the B3LYP/6-311+G(d,p) level (*R*^2^ = 0.98). The best-fit straight line is Equation 2 (red dashed line in Figure 1). Most of the absolute fitting residuals (85%) are less than 5 ppm, while the rest are within 11 ppm except the Al-phosphate species which has a large residual of 24 ppm. For the calculations using the 6-31G(d) basis set, the correlation between the B3LYP and HF models is also strongly linear (*R*^2^ = 0.99). Most of the fitting residuals are also within 5 ppm.

**Figure 2:**
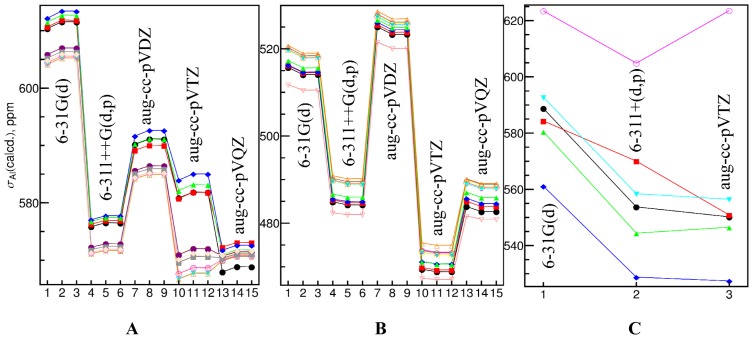
Calculated ^27^Al shielding constants using the GIAO method with the B3LYP functional with different basis sets in optimized geometries of [Al(OH_2_)_6_]^3+^ (**A**), [Al(OH)_4_]^−^ (**B**) and other complexes (**C**). The basis sets were labeled on the graphs. In Panels **A** and **B**, there are three data points for each basis set which represent the values calculated in vacuum, in the PCM environment of water and methanol. Twelve geometries in **A** were obtained at the B3LYP/aug-cc-pVQZ, B3LYP/aug-cc-pVTZ, B3LYP/aug-cc-pVDZ and B3LYP/6-311++G(d,p) levels in vacuum, in the PCM environment of methanol and water, and the data sets are colored in black, red, blue, green, cyan, magenta, yellow, brown, orange, pink, purple and gray, respectively. The last geometry in **A**, which is in light orange color, was obtained at the B3LYP/6-31G(d) level in the PCM environment of water. Ten geometries in **B** were obtained at the B3LYP/aug-cc-pVQZ, B3LYP/aug-cc-pVTZ, B3LYP/aug-cc-pVDZ and B3LYP/6-311++G(d,p) levels in vacuum, at the B3LYP/ aug-cc-pVQZ and B3LYP/aug-cc-pVTZ levels in the PCM environment of methanol, and at the B3LYP/aug-cc-pVQZ, B3LYP/aug-cc-pVTZ, B3LYP/aug-cc-pVDZ and B3LYP/ 6-31G(d) levels in the PCM environment of water, and the data sets are colored in black, red, blue, green, cyan, magenta, yellow, brown, orange and pink, respectively. Six complexes in **C** are [Al(oxalate)_3_]^3−^, [AlF_6_]^3−^, [Al(EDTA)]^−^, Al(lactate)_3_, [Al(malonate)_2_(OH_2_)_2_]^−^ and [Al(N≡CCH_3_)_6_]^3+^, respectively. All the values obtained with a same geometry are connected with straight lines.

A more comprehensive study including the calculations with Dunning’s correlation consistent basis sets [146,147], aug-cc-pV*X*Z (*X* = D, T and Q), was carried out for a limit number of complexes. The calculated shielding constants were shown in Figure 2. It is clear that the values did not converge well even at the aug-cc-pVTZ or aug-cc-pVQZ level for many geometries. However, the shielding constants calculated with different basis sets are highly correlated, which is consistent with the above observation. Jensen [16] proposed to use pcS-*n* basis sets with DFT methods to obtain the converged shielding constants. Here, we showed that even though there are large errors involved in the absolute shielding constants calculated with the commonly used basis sets, such as 6-31G(d) and 6-311+G(d,p), the errors are not random and a linear relationship exists between the values obtained with these small size basis sets and basis set limits. Therefore, these basis sets are still valuable in predicting ^27^Al chemical shifts.

The MP2 model has been considered a more accurate model in electronic structure calculations. We also calculated the chemical shielding constants using this model with the 6-311+G(d,p) basis set. Because the large memory cost required and CPU time needed for the model, the calculations were done only for three species, [Al(OH_2_)_6_]^3+^, [AlF_6_]^3−^ and [AlF_5_(OH_2_)]^2−^. The calculated chemical shielding constants are also strongly correlated with the values obtained at the B3LYP/6-311+G(d,p) level, *R*^2^ = 0.97. The slope of the best-fit equation is 0.98. The calculations indicate a trend, but more than three data sets (species) will have to be investigated before more firm quantitative relationships can be established.

### 2.2. Comparison between Experimental and Calculated Chemical Shifts

From the previous subsection, we see that the shielding constants have a strong linear correlation among five levels of theory, though the absolute numbers differ from each other significantly. In practice, the chemical shifts are our primary concern, since the absolute chemical shielding constants are difficult to measure experimentally. Chemical shifts are related to shielding constants through the approximate equation, δ_species_ = σ_ref_ – σ_species_. ^27^Al chemical shifts are usually referred to the aqueous [Al(OH_2_)_6_]^3+^ species in acidic solutions. We use the averaged chemical shielding constant of the [Al(OH_2_)_6_]^3+^ species at the corresponding level to calculate the chemical shifts of other species. The results are given in Table 1.

How do these models perform when compared with the experimental measurements? We searched the literature for experimental ^27^Al NMR studies of Al(III) species and picked the species whose ^27^Al chemical shifts were assigned. The measurements include both solution-state and solid-state magic-angle-spin (MAS) ^27^Al NMR spectra. For the latter, the isotropic chemical shifts were calculated by fitting the simulated spectra with the experimental spectra. The solution-state NMR measurements were for the Al(III) species in both aqueous and organic solvents. Therefore, the experimental chemical shifts are measured at a wide range of different chemical environments. But the calculations were done for the isolated molecules. How strongly are the ^27^Al chemical shifts dependent on the long-range chemical environment? The answer might be different for different species, but the environment effect might be small for most Al(III) species because the first coordination layer dominates the shielding effect to the ^27^Al nucleus. For example, the chemical shifts of the tetrahedral-coordinated [Al(OC(CF_3_)_3_)_4_]^−^ with various kinds of counter ions in different solvents, range from 38.8 to 33.8 ppm [140–143]. On the other hand, the chemical environment has a significant effect on the small [AlF_6_]^3−^ species and fast ligand exchange occurs in aqueous solutions [148,149]. The solid-state MAS NMR studies found the isotropic chemical shifts of [AlF_6_]^3−^ range from −0.1 to −17.9 ppm for which the counter ions include H^+^, K^+^, NH_4_^+^ and Rb^+^[150]. The values for [AlF_5_(OH_2_)]^2−^ obtained from the solid-state MAS NMR spectra range from −6.3 to −14.4 ppm [150]. These results reflect the sensitivity of chemical shifts of small size species to chemical environment. The environment could affect the ^27^Al chemical shifts through two aspects. The first is that the geometry parameters, such as bond lengths and bond angles, of the first coordination layer could be perturbed by the packing forces, by the electrical field and/or by solvation. The other factor is that the chemical environment other than the first coordination layer could contribute to the shielding or to the deshielding effect to the metal center. For the isolated molecule models used to compute the chemical shieldings, counter ions and solvents were not included. Therefore, only the first effect was accounted for in the calculations.

**Figure 3:**
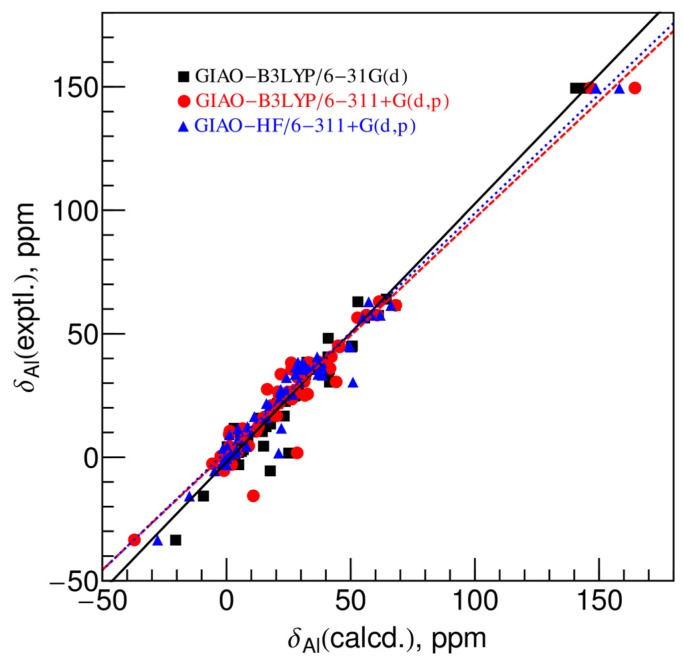
Correlation between the experimental ^27^Al chemical shifts *versus* the calculated values using the X-ray crystallographic geometries at the GIAO-B3LYP/6-31G(d) (black squares), the B3LYP/6-311+G(d,p) (red circles) and the HF/6-311+G(d,p) (blue triangles) levels. The black solid line, the red dashed line and the dotted blue lines are the best-fit linear regression of the three data sets, respectively, and the best-fit equations are given in Equations 3–5.

The calculated chemical shifts *vs.* the experimental chemical shifts are shown in Figure 3. Black squares are the calculated shifts at the GIAO-B3LYP/6-31G(d) level, and red circles and blue triangles are the calculated values at GIAO-B3LYP/6-311+G(d,p) and GIAO-HF/6-311+G(d,p), respectively.

Comparing the calculated chemical shifts at the GIAO-B3LYP/6-31G(d) level with the experimental values, the absolute errors for most species are less than 12 ppm except for three outliers. About one third (31%) of the absolute errors are less than 2 ppm, while more than 79% in total are less than 6 ppm. The overall root-mean-square difference (RMSD) is 6.5 ppm. If the three outliers whose absolute errors are larger than 12 ppm are removed, the RMSD decreases to 4.4 ppm. From this result, we see that the popular GIAO-B3LYP model with the 6-31G(d) basis set performs quite well for predicting the ^27^Al chemical shifts using the X-ray measured geometries.

The three outliers are [AlF_6_]^3−^, [Al(NCCH_3_)_6_]^3+^ and [Al_4_(μ_2_-OH)_2_(μ_2_-H_−1_malato)_4_]^2−^. In calculating δ_Al_ for [AlF_6_]^3^ the large error is probably due to its small size [150]. Calculations including the ion’s environment beyond the binding ligands would probably significantly improve the result. For the acetonitrile complex, the ligand is disordered in the crystals and not accounting for the distribution in geometries might lead to a significant error [118]. For the polymeric malate complex, the ^27^Al NMR spectra are not well defined, so there might be a significant error associated with the experimental determination of δ_Al_[73].

(3) $$\delta_{\text{A}1}\;(\text{GIAO}-\text{B}3\text{LYP}/6-31\text{G}(\text{d}))=-1.18+1.03\;\delta_{\text{A}1}(\text{expt}),R^{2}=0.94$$

(4) $$\delta_{\text{A}1}\;(\text{GIAO}-\text{B}3\text{LYP}/6-311+\text{G}(\text{d},\text{p}))=1.67+0.95\;\delta_{\text{A}1}(\text{expt}),R^{2}=0.94$$

(5) $$\delta_{\text{A}1}\;(\text{GIAO}-\text{HF}/6-311+\text{G}(\text{d},\text{p}))=2.63+0.96\;\delta_{\text{A}1}(\text{expt}),R^{2}=0.96$$

When the larger basis set, 6-311+G(d,p), is used, the accuracy improves slightly. The RMSD between the calculated chemical shifts at the GIAO-B3LYP/6-311+G(d,p) level and the experimental values for all the species is 6.6 ppm and it decreases to 3.9 ppm after removing the three outliers whose absolute errors are larger than 12 ppm. About 40% of the absolute errors are less than 2 ppm, while 80% in total are less than 6 ppm.

The accuracy of the chemical shifts calculated at the GIAO-HF/6-311+G(d,p) level is slightly worse than that of the GIAO-B3LYP/6-31G(d) level. Although the RMSD for all the data points is 5.7 ppm which is less than the other two models, it decreases to 4.8 ppm after removing two outliers whose absolute errors are larger than 12 ppm. One third (33%) of the absolute errors are less than 2 ppm and 72% in total are less than 6 ppm.

By comparing the results of the three levels of theory, we see that they are similar in terms of the capability in predicting the ^27^Al chemical shifts, though the GIAO-B3LYP/6-311+G(d,p) model is slightly better than the other two.

As seen from Figure 3, the calculated chemical shifts at three levels were well correlated with the experimental results, except for a few outliers. The best-fit equations are given in Equations 3~5 for the correlation between the calculated chemical shifts at the GIAO-B3LYP/6-31G(d), GIAO-B3LYP/6-311+G(d,p) and GIAO-HF/6-311+G(d,p) levels, respectively, *versus* the experimental values (black solid line, red dashed line and blue dotted line, respectively in Figure 3).

### 2.3. Geometry Optimization

In a practical application scenario, the structure of the proposed complex is unknown. Geometry optimization is used to obtain the structure in the first place. Does the error of the calculated geometries contribute significantly to an error in predicting ^27^Al chemical shifts? To investigate this question, the experimental geometries of 33 complexes were optimized at the B3LYP/6-31G(d) level, followed by a shielding tensor calculation using the GIAO-B3LYP/6-31G(d) model.

**Figure 4:**
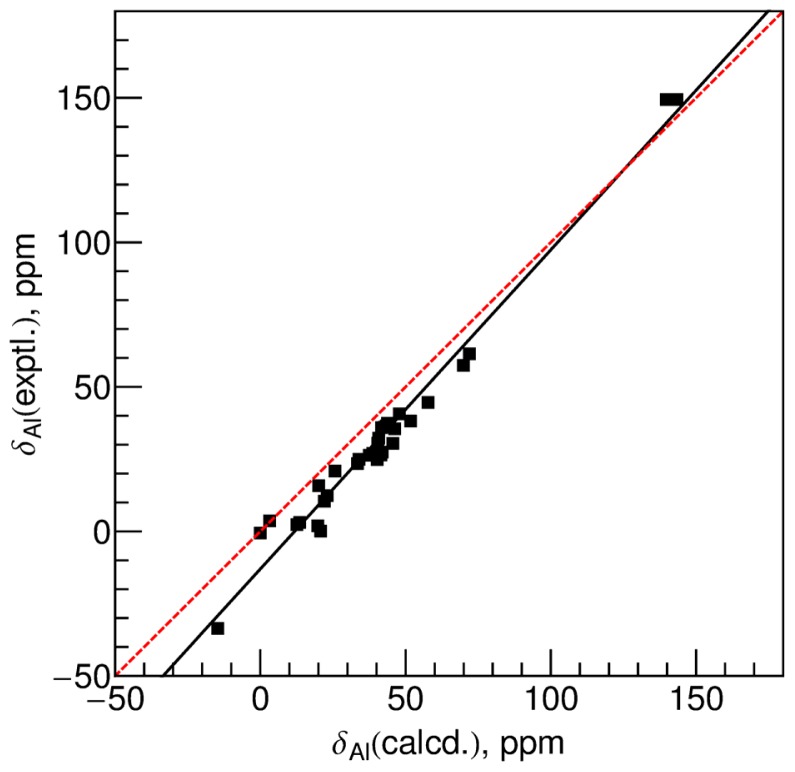
Correlation between the experimental ^27^Al chemical shifts *versus* the calculated values at the GIAO-B3LYP/6-31G(d) level using the optimized geometries at the B3LYP/6-31G(d) level. The black solid line is the best-fit linear regression of the data and the best-fit equation is given in Equation 6, while the dashed red line is the ideal diagonal line (*y* = *x*).

The calculated δ_Al_ are plotted against the experimental values in Figure 4. On one hand, the calculated δ_Al_ are systematically larger than the experimental values; on the other hand, the slope of the best-fit equation which is given in Equation 6 and plotted as the black line in Figure 4 is 1.10. The 95% confidence interval for the slope is 1.04 to 1.17. The systematic larger calculated chemical shifts may be because the shielding constant of the reference [Al(OH_2_)_6_]^3+^ species is overestimated in the optimized geometry. The calculated σ_Al_ in the optimized geometry is 16.7 ppm larger than that in the X-ray geometry, which reflects the elongation of Al–O bonds by 0.06 Å in the optimized geometry compared with the mean value in the X-ray geometry. The latter deviation reflects that the single-molecule calculated geometry at the B3LYP/6-31G(d) level causes significant errors in predicting δ_Al_. Until such time as experimental techniques can unequivocally determine structures of molecules in liquid solvents, this issue cannot be unambiguously settled.

(6) $$\delta_{\text{A}1}\;(\text{GIAO}-\text{B}3\text{LYP}/6-31\text{G}(\text{d})/\text{B}3\text{LYP}/6-31\text{G}(\text{d}))=-13.0+1.10\;\delta_{\text{A}1}\;(\text{expt}),R^{2}=0.97$$

The RMSD for all the data points is 10.9 ppm. Even after removing the points with errors larger than 12 ppm, the RMSD is about 8.4 ppm. If we offset all the calculated δ_Al_ by 15.4 ppm, *i.e.* to let the best-fit equation cross the origin point, the calculated RMSD is still as large as 7.7 ppm. Therefore, an accurate geometry is critical in calculating ^27^Al chemical shifts.

### 2.4. Solvent Effect

Solvent effect plays an important role in solution-state ^27^Al NMR. Without the constraint of the periodic boundary condition in the crystals, the geometries will be slightly different, the long-range interactions will be dramatically different and dynamical effect will be also different. In this study, we only examined how the commonly used implicit solvation model affects the prediction of ^27^Al chemical shifts. The self-consistent reaction field (SCRF) method [151] with the polarizable continuum model (PCM) [152] of water and/or methanol was applied to the calculation of the shielding constants and the geometry optimization. In Figure 2, we have seen that the calculated shielding constants of [Al(OH_2_)_6_]^3+^ and [Al(OH)_4_]^−^ in the PCM environment of water or methanol are nearly the same as those obtained in vacuum using the same geometry. However, the calculated shielding constants using the geometries optimized in the PCM environment of water or methanol differ by 5~10 ppm from those obtained with the optimized geometries in vacuum. The differences in [Al(OH_2_)_6_]^3+^ are larger than those in [Al(OH)_4_]^−^, which reflects the former species is more sensitive to the solvation effect. At the B3LYP/aug-cc-pVQZ level, the calculated Al–O bond length of [Al(OH_2_)_6_]^3+^ in water or methanol is 0.036 Å shorter than that obtained in vacuum in average (1.896 Å *vs.* 1.932 Å), while the difference is only 0.008 Å in [Al(OH)_4_]^−^ (1.770 Å *vs.* 1.778 Å).

**Figure 5:**
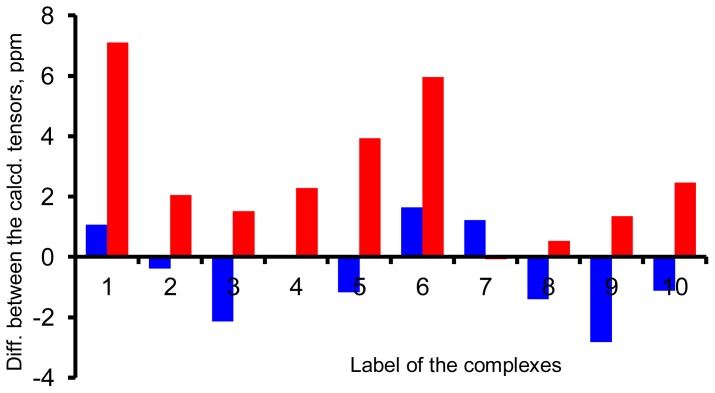
Difference between the calculated σ_Al_ for 10 Al(III) complexes. The blue bars are the differences between the values in the PCM environment of water and in vacuum using the X-ray geometries. The red bars are the differences between the values calculated using the geometries optimized at the B3LYP/6-31G(d) level in the PCM environment of water and those calculated using the X-ray geometries in vacuum. All the σ_Al_ are calculated at the GIAO-B3LYP/6-31G(d) level. The complexes are [Al(OH_2_)_6_]^3+^, [Al(oxalate)_3_]^3−^, [AlF_6_]^3−^, Al(8-hydroxyquinoline)_3_, [Al(EDTA)]^−^, Al(lactate)_3_, [Al(malonate)_2_(OH_2_)_2_]^−^, [Al(H_−1_citrate)_2_]^5−^, [Al(N≡CCH_3_)_6_]^3+^ and Al(maltol)_3_ for 1–10, respectively.

Two more groups of calculations were carried out on 10 representative complexes. In the first group of calculations, the influence of the PCM model to the calculation of the shielding constants was examined using the X-ray geometries at the GIAO-B3LYP/6-31G(d) level. In the second group of calculations, both the geometry optimization and the shielding constants calculation were carried out in the PCM environment of water at the B3LYP/6-31G(d) level. The calculated σ_Al_ values were compared with the values obtained in vacuum using the X-ray geometries. The differences are plotted in Figure 5, where the blue and red bars are for the first and second groups of calculations, respectively. Again, we see that the PCM model only causes a minor change to the calculated σ_Al_ if the same geometry was used. However, if the geometry optimization was applied, significant changes were observed. This result again says that the geometry parameters of the complexes have a big influence on the calculated σ_Al_.

## 3. Computational Details

The crystal structures of 114 Al(III) complexes, determined by single-crystal X-ray diffraction, were obtained from the Cambridge Crystal Structural Database (Cambridge Crystallographic Data Centre, Cambridge, UK). They were used to build the molecular structures of the species. If there was more than one Al(III) species in the asymmetric unit, a molecular structure was built for each structure. For the disordered crystal structures, only the structure with major population was used to build the molecular model. In total, 213 molecular models were created from 206 crystal structures for 114 species. 33 complexes of small to intermediate sizes were optimized using the X-ray structures as the starting point.

Only a single molecule was included in each molecular model. Hydrogen positions were put in their ideal positions with the aid of GaussView 3.0 (Gaussian, Inc., Wallingford, CT, USA) if they were missing from the crystal structures. All the counter ions and solvent molecules were excluded from the models. Chemical shielding tensors were calculated for these X-ray geometry models and the models with further geometry optimization at the B3LYP/6-31G(d) level.

All electronic structure calculations were done with the Gaussian03 program suite [153]. Chemical shielding tensors were calculated with the gauge-invariant atomic orbital (GIAO) formalism [5,6] of three theory models, Hartree-Fock, Becke-Lee-Yang three-parameter hybrid density functional theory (B3LYP) [154–157] and Møller-Plesset second-order perturbation theory (MP2) [158], with a series of basis sets, 6-31G(d) [159], 6-311+G(d,p) and 6-311++G(d,p) [160] and aug-cc-pV*X*Z (*X* = D, T and Q) [146,147]. The implicit solvent model of water and methanol was calculated using the self-consistent reaction field (SCRF) method [151] with the polarizable continuum model (PCM) [152]. ^27^Al chemical shifts were obtained by subtracting the isotropic chemical shielding constants from the mean chemical shielding constant of the reference species, [Al(OH_2_)_6_]^3+^, calculated at the same theory level.

## 4. Conclusions

The main purpose of this work is to give a quantitative evaluation of the errors involved in the calculation of ^27^Al chemical shifts. We differentiated the source of the errors due to the limitations of the computational models in calculating the chemical shielding tensors and the inaccuracy in geometry prediction by using the X-ray measured geometries and the calculated geometries to compute chemical shifts. The RMSD between the calculated shifts and the experimental values is approximately 5 ppm if the X-ray crystallographic structures were used, while the RMSD is more than 10 ppm for the optimized geometries at the B3LYP/6-31G(d) level. Although we could not isolate the error due to the structural uncertainties in the X-ray crystallography, the popular GIAO-B3LYP/6-31G(d) model produces quite accurate chemical shifts.

There are other error sources not considered in this study, such as the quality of DFT functionals and dynamical effects [161]. In addition, the structure of a complex in solution would be different from its crystal structure; the solvent effect is also more complicated than what has been studied here. However, the results presented in this study shows that the error associated with the shielding constants calculation is small compared with the error in the prediction of geometry. In particular, Jensen showed that the error in the shielding constants calculation can be controlled to less than 1 ppm. In the future, we should focus on predicting a more accurate geometry in solution or an ensemble of structural configurations generated using molecular dynamics.

## Figures and Tables

**Table 1:** Comparison between the calculated and experimental ^27^Al chemical shifts.

Species a	Refs.	^27^Al chemical shifts				

		GIAOB3LYP/ 6-31G(d) b	GIAOB3LYP/ 6-311+G(d,p) b	GIAOHF/6-311+ G(d,p) b	GIAO-B3LYP/6-31G(d)//B3LYP/6-31G(d) c	Expt.^d^
**Chemical shift reference**						

[Al(OH_2_)_6_]^3+^	[20–24]	0 ± 2.87	0 ± 3.44	0 ± 2.99	0	0

**Al(III) hydrolysis products**						

[Al_2_(μ_2_-OH)_2_(OH_2_)_8_]^4+^	[25]	0.74 ± 1.94	1.73 ± 0.11	−1.30 ± 2.35	3.23	4.2

[Al_8_(μ_3_-OH)_2_(μ_2_-OH)_12_(OH_2_)_16_]^10+^	[26]	0.34 ± 0.39				4.8 (MAS)
		5.67 ± 0.11				8.4
		9.53 ± 0.10				11

[AlO_4_Al_12_(μ_2_-OH)_24_(OH_2_)_12_]^7+^ (Keggin-Al_13_)	[25]	52.95 ± 0.02	61.50 ± 0.52	57.30		63.5
		7.05 ± 0.53	6.53 ± 0.67	4.52 ± 0.65		12

[Al_13_(μ_3_-OH)_6_(μ_2_-OH)_12_(OH_2_)_6_]^15+^ (flat-Al_13_)	[27]	15.74				
		9.71 ± 0.79				
		0.25 ± 0.32				

[Al_30_(μ_4_-O)_8_(μ_3_-OH)_6_(μ_2_-OH)_50_(OH_2_)_24_]^15+^ (Al_30_)	[25]	4.08 ± 1.18				4
		8.57 ± 0.41				5~12
		64.38 ± 0.51				64.5

**Al(III) complexation with carboxylate and carbonyl groups**						

[AlL_6_]^3−^, L = formate	[28]	0.92	−4.12	−2.56		

[AlL_6_]^3+^, L = dimethylformamide	[29,30]	3.20 ± 0.46	−0.78 ± 0.60	0.42 ± 0.41		

[AlL_6_]^3+^, L = dimethylacetamide	[31]	−6.97	−16.13	−15.50		

[AlL_3_]^3−^, L = oxalate	[32–39]	13.35 ± 0.97	14.77 ± 0.97	15.43 ± 1.12	20.15	16.3

[AlL_2_(OH_2_)_2_]^−^, L = malonate	[40]	4.81	4.32	3.65	19.77	2.5

[AlL_2_(OH_2_)_2_]^−^, L = methylmalonate	[21]	8.29	2.93	4.14		

[AlL_3_]^3+^, L = acetylacetone	[41–44]	2.44 ± 0.84	−2.29 ± 0.50	−0.40 ± 0.87		0.88

[AlL_3_]^3+^, L = 1,3-diphenylpropane-1,3-dione	[45]	7.39	−0.37	2.15		

[AlL_3_]^3+^, L = 1,3-dimesitylpropane-1,3-dione	[46]	5.26	−0.38	1.65		

[AlL_3_]^3+^, L = 1,3-bis(pentafluorophenyl)propane-1, 3-dione	[47]	2.39	−1.52	0.37		

[AlL_3_]^3+^, L = methylacetoacetate	[48]	6.54	2.60	2.60		

[AlL_3_]^3+^, L = *t*-butylacetoacetate	[49]	3.74	−0.91	−1.17		

[AlL_3_]^3+^, L = 3-(4-pyridyl)-2,4-pentanedione	[50]	2.17	−3.12	−0.40		

[AlL_3_]^3+^, L = 3-phenyl-2,4-pentanedione	[50]	2.53	−3	−0.51		

AlL_3_, L = *iso*-maltol	[51]	5.98	1.22	1.37	12.60	2.9

**Al(III) complexation with catechol-like ligands**						

AlL_3_, L = maltol	[52]	34.10 ± 0.17	39.04 ± 0.13	31.82 ± 0.12	43.36	37, 38

AlL_3_, L = 2-ethylmaltol	[53]	33.86	38.80	31.48	43.74	38

[AlL]^3−^, L = tris(*N*,*N*′-diethyl-2,3-dihydroxoterephthalamide)diamine	[54]	30.30	32.66	26.75		26

AlL_3_, L = 3-hydroxy-2-pyridone	[55]	24.09	33.43	27.05		

AlL_3_, L = 1,2-dimethyl-3-hydroxy-4-pyridone	[56,57]	32.32 ± 0.00	37.04 ± 0.00	28.75 ± 0.00		36, 39

AlL_3_, L = 1-*n*-propyl-2-methyl-3-hydroxy-4-pyridone	[58]	31.80	35.76	28.37		37

AlL_3_, L = 1-*n*-butyl-2-methyl-3-hydroxy-4-pyridone	[58]	31.21	35.51	27.77		37

AlL_3_, L = 1-*p*-tolyl-2-methyl-3-hydroxy-4-pyridone	[59]	33.74	38.55	30.39		37

AlL_3_, L = tropolone	[60]	32.68	36.11	29.72	42.72	36.6

AlL_3_, L = 6-*i*-propyltropolone	[61]	33.42	36.50	29.84		

Al_2_L_3_, L = 1,3-bis((3-oxy-1-methyl-2-oxo-1,2-dihydropyridin- 4-yl)carboxamido)-2,2-dimethylpropane	[62]	28.67 ± 0.00	34.18 ± 0.00			

[Al_2_(μ_2_-O)_2_ L_4_]^4−^, L =1,2-dihydroxyanthraquinone	[63]	23.01 ± 0.00				

[Al_2_(μ_2_-O)_2_ L_4_]^4−^, L =1,2,4-trihydroxyanthraquinone	[64]	23.67 ± 0.05				23.1

**Al(III) complexation with carboxylate and hydroxyl groups**						

*mer*-[AlL_3_]^3−^, L = glycolate	[65]	30.86	37.08	28.13		

*fac*-[AlL_3_]^3−^, L = glycolate	[66]	27.65	31.59	23.55	34.00	25.5

[AlL_3_]^3−^, L = lacate	[67]	23.12 ± 0.13	26.32 ± 0.26	21.16 ± 0.16	33.44	24

[AlL_2_]^5−^, L = H_−1_citrate	[68,69]	19.07 ± 0.45	18.04 ± 0.68	17.33 ± 0.71	25.69	21.4

[Al_2_L_3_]^6−^, L = H_−1_citrate	[70]	25.36	20.72	17.98		
		20.28	14.44	11.40		

[Al_3_L_3_(*μ*_2_-OH)(OH_2_)]^4−^, L = H_−1_citrate	[71]	3.43	−0.44	0.05	20.74	0.6
		14.42	9.81	9.32	22.00	10.9
		15.90	9.81	8.41	23.00	12.9

[Al_3_L_2_(μ_2_-OH)_2_(OH_2_)_4_]^−^, L = H_−1_citrate	[20]	5.43 ± 0.70				
		25.75				

[Be_2_Al_2_L_4_]^6−^, L = H_−1_citrate	[72]	17.79 ± 0.87				14.0

[Be_6_Al_6_L_6_]^18−^, L = H_−1_citrate	[72]	3.07 ± 0.18				12.2

[Al_4_L_6_]^6−^, L = H_−1_malate	[73]	23.27 ± 0.00	20.20 ± 0.00	15.83 ± 0.00		17.2
		6.35 ± 0.00	1.14 ± 0.00	1.18 ± 0.00		1.6

[Al_4_L_4_(μ_2_-OH)_2_]^2−^, L = H_−1_malate	[73]	25.10 ± 0.79	28.46 ± 0.87	21.02 ± 1.06		2.2,1.6
		5.59 ± 1.02	1.83 ± 1.28	1.80 ± 1.11		1.1

[Al_2_L_2_(OH_2_)_2_]^2−^, L = H_−1_saccharate	[74]	28.61 ± 0.00	29.21 ± 0.00	24.36 ± 0.00	65.49	

[Al_6_L_4_(μ_2_-OH)_8_]^6−^, L = H_−4_galactarate	[75]	16.43 ± 2.40	17.82 ± 1.13	11.64 ± 1.12		16.1, 16.8, 17.4, 17.7 (MAS)
		20.54 ± 1.74	21.27 ± 1.14	15.86 ± 1.75		22.6, 21.9 (MAS)

Al_2_L_2_(μ2-*t*-butoxo)2(*t*-butyoxy-*t*-butylperoxy), L = *o*-methoxycarbonyl phenoxy	[76,77]	11.91 ± 0.00	3.99 ± 0.00	3.94 ± 0.01		
		62.46 ± 0.00	61.47 ± 0.01	64.46 ± 0.01		

[AlL_2_L′_2_]^−^, L = acetylacetonate, L′ = *iso*-propoxo	[78]	9.25	2.89	4.44		

Al_2_L_4_L′_2_, L = 3,5-heptanedionato, L′ = *iso*-propoxo	[79]	9.69 ± 0.00	4.71 ± 0.00	2.40 ± 0.00		

[AlL]^+^, L = (2-hydroxybenzoyl-2-aminoethyl)-bis (2,3-dihydroxybenzoyl-2-aminoethyl)ammonio	[80]	8.05	1.26	3.95		

[AlL]^+^, L = tris((2-Hydroxybenzoyl)-2-aminoethyl)ammine	[81]	7.04	0.38	2.64		

[AlL]^+^, L = tris((2-Hydroxy-3-methoxybenzoyl)-2- aminoethyl)ammine	[81]	6.05	0.27	2.24		

**Al(III) complexation with polyamino-polycarboxylates**						

[AlL]^−^, L = ethylenediaminetetraacetate	[82–84]	40.87 ± 2.57	42.22 ± 1.67	36.49 ± 1.27	47.88	41.2

[AlL_2_]^−^, L = iminodiacetate	[85–87]	37.94 ± 0.66	41.68 ± 0.60	35.15 ± 0.42	41.69	36.5

[AlL_2_]^−^, L = methyliminodiacetate	[88]	41.69 ± 0.62	43.18 ± 0.91	36.82 ± 0.63		

Al_2_L_2_(μ_2_-OH)_2_(OH_2_)_2_, L = iminodiacetate	[86,89]	20.12 ± 0.67	22.07 ± 1.60	18.56 ± 1.03		

AlL, L = 1,4,7-triazacyclononane-N,N′,N″-triacetate	[90,91]	52.17 ± 0.82	49.37 ± 1.44	44.68 ± 0.73		

[AlL_2_]^−^, L = dipicolinate	[92]	22.62	27.33	23.22		

AlL(OH_2_)_2_, L = nitrilotriacetate	[93]	24.77	25.74	21.60		

[Al_2_L_2_(μ_2_-OH)_2_]^2−^, L = nitrilotriacetate	[93]	27.42 ± 0.00	26.82 ± 0.00	22.16 ± 0.00	40.27	25.4

Al_2_L_2_(OH)_2_, L = *N*-(2-oxyethyl)iminodiacetate	[94]	30.05 ± 0.00	28.59 ± 0.00	24.17 ± 0.00		32.8

Al_2_L_2_(μ_2_-OH)_2_, L = ethylene-*N*,*N*′-bis(3-hydroxy propionato)	[95]	29.00 ± 1.69	27.50 ± 1.33	24.23 ± 1.41	38.74	25~30

[Al_4_L_2_(μ_2_-OH)_4_]^2−^, L = 1,3-diamino-2-propanolato-*N*,*N*,*N*′,*N*′-tetraacetato	[86]	27.26 ± 1.69	25.83 ± 1.00	19.69 ± 0.56		

[Al_4_L_2_L′_2_(μ_2_-OH)(μ_2_-O)]^−^, L = *N*-(3-ammoniopropyl)carbamato, L′ = 1,3-diamino-2-propanolato- *N*,*N*,*N*′,*N*′-tetraacetato	[86]	30.00 ± 0.50	27.11 ± 1.36	22.54 ± 0.15		

**Al(III) complexation with mixed O and N ligands**						

AlL_3_, L = 8-hydroxyquinoline	[96–100]	30.68 ± 1.84	31.27 ± 1.37	29.39 ± 1.68	40.41	31.1

AlL_3_, L = 2-methyl-8-hydroxyquinoline	[101]	33.28	35.11	31.33		

AlL_2_L′, L = 2-methyl-8-hydroxyquinoline, L′ = picolinato	[102]	28.81	29.78	26.97		

Al_2_L_4_(μ_2_-2-(ethoxy)ethoxo))_2_, L = 8-hydroxyquinoline	[103]	28.14 ± 0.44	24.82 ± 1.21	22.39 ± 1.29	37.52	26.9

Al_2_L_3_, L = 1,3-bis(8-hydroxyquinolin-7-yl)-2-methylenepropane	[104]	33.55 ± 0.00	34.88 ± 0.00	31.92 ± 0.00		

AlL_3_, L = 2-(2-hydroxyphenyl)-5-phenyl-1,3,4-oxadiazole	[105]	8.95	−0.96	3.41		

[AlL_2_]^−^, L = 1-phenyl-3,5-bis(2-oxyphenyl)-1,2,4-triazole	[106]	6.91	0.85	2.80		

[AlL_2_(OH_2_)_2_]^+^, L = *N*,*N*′-bis(3,5-*di*-*t*butylsalicylidene) ethylenediamine	[107]	7.41	5.02	8.07		

[AlL_2_(CH_3_OH)_2_]^+^, L = *N*,*N*′-bis(3,5-*di*-*t*butylsalicylidene) ethylenediamine	[107]	11.62 ± 1.47	8.59 ± 2.08	9.40 ± 1.21		

[AlL_2_(OH_2_)_2_]^−^, L = 1,9-bis(2-oxyphenyl)-5-phenyldipyrrine	[108]	6.67	3.16	5.88		

AlLL′_2_, L = 5,10,15,20-tetraphenylporphyrine, L′ = tetrahydrofuran	[109]	−5.41	−15.23	−10.73		

AlL_2_, L = 3,5-*di*-*t*-butyl-1,2-quinone-1-(2-hydroxy-3,5-*di*-*t*butylphenyl) imine	[110]	50.61	50.41	41.14		

AlL_2_L′_2_, L =2,2,6,6-tetramethylheptane-3,5-dionato, L′ = 2-((2-hydroxy)benzylidene)amino)phenolato	[111]	12.17 ± 0.00	7.15 ± 0.00	6.82 ± 0.00		

Al_2_L_4_(μ_2_-OH)_2_, L = 2-(2-oxyphenyl)benzimidazole	[112]	7.96 ± 0.00	1.56 ± 0.00	2.80 ± 0.00		

Al_2_L_4_(μ_2_-OH)_2_, L = 2-(2′-hydroxyphenyl)-2-benzoxazolato	[113]	14.92 ± 0.21	± 0.00	8.12 ± 0.05		5

[AlL_2_]^−^, L = nordesferriferrithiocin	[114]	19.95	17.46	16.86		

[AlL_2_]^−^, L = desferriferrithiocin	[114]	12.84	4.89	7.58		

AlL, L = alumichrome A	[115]	26.96				31.54

[AlL]^+^, L =tris(5′-chloro-2′-hydroxybenzylaminoethyl)amine	[116]	19.87	11.25	13.95		

[Al_3_(μ_3_-*O*)L_6_L′_3_]^+^, L = acetate, L′ = acetonitrile	[116]	2.66 ± 0.24	−1.08 ± 0.17	−0.16 ± 0.57		

**Al(III) complexation with N's**						

[AlL_6_]^3+^, L = acetonitrile	[118]	−20.44 ± 2.21	−37.13 ± 1.12	−27.74 ± 1.59	−14.60	−33

AlL_3_, L = bis(2-pyridyl)amido	[119]	28.36	20.50	23.19		

AlL_3_, L = 1,3-bis(2-methylphenyl)triazenido	[120]	28.62	16.35	21.86	41.93	28

AlL_3_, L = 1,3-bis(4-chlorophenyl)triazenido	[120]	28.62	23.05	23.15		26

AlL_3_, L = 1,3-bis(4-methoxyphenyl)triazenido	[120]	26.60	20.75	21.82	41.48	27

AlL_3_, L = 1,3-diphenyltriazenido	[121]	26.72	21.26	21.82		25

**Al(III) complexation with P and other ligands**						

[Al(H_2_L)_3_(HL)_3_]^6−^, L = phosphate	[122]	−9.22	10.78	−14.88		−15.2

[Al_4_(HL)_4_L′_12_]^4+^, L = phosphate, L′ = ethanol	[123]	1.32 ± 0.00				−8.7, −8.8 (MAS)
		1.77 ± 0.00				−8.2

[Al_2_L_2_L′_8_]^4+^, L = phenylphosphinato, L′ = *n*-butanol	[124]	4.91 ± 0.00	2.04 ± 0.00	0.09 ± 0.00		−2.5

[Al_3_L_2_L′_10_(μ_2_-OH)]^4+^, L = phenylphosphinato, L′ = ethanol	[125]	−0.02				−2.2
		3.42 ± 0.69				1.5

[Al_5_L_4_L′_10_(μ_2_-OH)_2_]^5+^, L = phenylphosphinato, L′ = *sec*-butanol	[126]	−0.32 ± 0.07				0
		40.99				48.7

**Al(III)-fluoride complexes**						

[AlF_6_]^3−^	[127–132]	17.60 ± 0.84	−1.05 ± 0.88	−4.77 ± 1.22		−5, −0.1, −0.6, −2.4, −2.7, −2.8, −17.9,

[AlF_5_(OH_2_)]^2−^	[129, 133–135]	17.46 ± 1.26	5.18 ± 1.46	−0.05 ± 2.33		−6.3, −12.0, −13.8, −14.4

[Al_2_F_10_]^4−^	[136,137]	18.35 ± 0.97				

[Al_4_F_18_]^6−^	[129]	12.62 ± 0.06				

[Al_7_F_30_]^9−^	[138]	−3.59				
		12.16 ± 0.29				

[AlF_2_L_4_]^+^, L = pyridine	[139]	6.74 ± 0.47	2.10 ± 1.75	4.19 ± 0.04	13.53	3.6 (MAS)

**Tetrahedral-coordinated Al(III) with O's**						

[Al(OR)_4_]^−^, R = C(CF_3_)_3_	[140–143]	40.55	33.09	38.36	51.71	38.8
		41.41	33.35	38.80		36.2
		39.71	32.22	36.79		37.5
		36.58				
		35.84	26.16	32.88		36
		41.08	29.83	38.56		33.8
		31.80	21.82	27.67		34.1
		39.66	31.83	36.86		34.1
		41.91		39.54		

[Al(OR)_4_]^−^, R = CH(CF_3_)_2_	[142,144]	58.23 ± 0.33	56.30 ± 0.11	58.42 ± 0.64	69.97	58
		61.18	60.69	61.91		58
		55.61	52.74	54.95		56.9

[Al(OR)_4_]^−^, R = C(CH_3_)(CF_3_)_2_	[142,144]	50.29	45.58	49.88		45.2
		50.71	44.95	49.34		45.6

[(OR)_3_Al FAl(OR)_3_]^−^, R = C(CF_3_)_3_	[141]	32.62 ± 1.47	27.89 ± 1.89	33.22 ± 2.21	46.30	36

**Tetrahedral-coordinated Al(III) with C's and N's**						

Al_2_L_2_L′_4_, L = pyrazolyl, L′ = *t*-butyl	[145]	143.64 ± 0.00	146.56 ± 0.00	148.74 ± 0.00	143.42	150

Al_2_L_2_L′_4_, L = 3,5-dimethylpyrazolyl, L′ = methyl	[77]	140.62 ± 0.00	164.46 ± 0.00	158.22 ± 0.00	139.69	150

AlLL′_2_, L = 1,3-diphenyltriazenido, L′ = *iso*-butyl	[120]	170.36	189.01	178.95		

AlLL′_2_, L = 1,3-diphenyltriazenido, L′ = 2,6-di-*t*-butyl-4-methylphenoxy	[120]	41.52	44.09	50.88	45.65	31

**Penta-coordinated Al(III) complexes**						

[AlL]^2−^, L = (2-hydroxy-3-methoxybenzoyl-2-aminoethyl)-bis(2,3-dihydroxybenzoyl-2-aminoethyl)amine	[79]	51.30	53.08			

[AlL]^2−^, L = (2-hydroxybenzoyl-2-aminoethyl)-bis(2,3-dihydroxybenzoyl-2-aminoethyl)amine	[79]	56.30				

AlLL′, L = *N*,*N*′-ethylene-bis(salicylideneamino-), L′ = methyl	[119]	61.12	66.86	64.69		

B_2_Al_2_(μ_2_-ethoxo)L_4_L′_6_, L = pyrazolyl, L′ = ethyl	[77]	67.85 ± 0.00	68.09 ± 0.00	66.20 ± 0.00	72.01	62

a “H_−1_” in the complexation species refers that the hydroxyl group or one of the hydroxyl groups in the ligand is deprotonated;

b The chemical shifts were obtained by subtracting the calculated isotropic chemical shielding constants from the averaged isotropic shielding tensor of the reference species computed at the same theoretical level. The calculated ^27^Al chemical tensors were obtained with the GIAO method at three theoretical levels: B3LYP/6-31G(d), B3LYP/6-311+G(d,p) and HF/6-311+G(d,p) and the X-ray crystallographic structures were used for the molecular geometries. The values following “±” are the standard deviations of the calculated values of either several models or multiple chemically equivalent sites;

c The molecular geometries were optimized at the B3LYP/6-31G(d) level, followed by a chemical shielding tensor calculation at the GIAO-B3LYP/6-31G(d) level;

d The values followed by “(MAS)” refer that the measurements were done using the solid-state MAS ^27^Al NMR spectroscopy and the isotropic chemical shifts were obtained by a fitting procedure from the spectra. Otherwise, the measurements were done in the solution state.
